# Sour Taste Responses in Mice Lacking PKD Channels

**DOI:** 10.1371/journal.pone.0020007

**Published:** 2011-05-19

**Authors:** Nao Horio, Ryusuke Yoshida, Keiko Yasumatsu, Yuchio Yanagawa, Yoshiro Ishimaru, Hiroaki Matsunami, Yuzo Ninomiya

**Affiliations:** 1 Section of Oral Neuroscience, Graduate School of Dental Science, Kyushu University, Fukuoka, Japan; 2 Department of Oral Physiology, Asahi University School of Dentistry, Gifu, Japan; 3 Department of Genetic and Behavioral Neuroscience, Gunma University Graduate School of Medicine, Maebashi, Japan; 4 JST, CREST, Tokyo, Japan; 5 Department of Applied Biological Chemistry, Graduate School of Agricultural and Life Sciences, The University of Tokyo, Tokyo, Japan; 6 Department of Molecular Genetics and Microbiology, Duke University Medical Center, Durham, North Carolina, United States of America; German Institute for Human Nutrition, Germany

## Abstract

**Background:**

The polycystic kidney disease-like ion channel PKD2L1 and its associated partner PKD1L3 are potential candidates for sour taste receptors. PKD2L1 is expressed in type III taste cells that respond to sour stimuli and genetic elimination of cells expressing PKD2L1 substantially reduces chorda tympani nerve responses to sour taste stimuli. However, the contribution of PKD2L1 and PKD1L3 to sour taste responses remains unclear.

**Methodology/Principal Findings:**

We made mice lacking PKD2L1 and/or PKD1L3 gene and investigated whole nerve responses to taste stimuli in the chorda tympani or the glossopharyngeal nerve and taste responses in type III taste cells. In mice lacking PKD2L1 gene, chorda tympani nerve responses to sour, but not sweet, salty, bitter, and umami tastants were reduced by 25–45% compared with those in wild type mice. In contrast, chorda tympani nerve responses in PKD1L3 knock-out mice and glossopharyngeal nerve responses in single- and double-knock-out mice were similar to those in wild type mice. Sour taste responses of type III fungiform taste cells (GAD67-expressing taste cells) were also reduced by 25–45% by elimination of PKD2L1.

**Conclusions/Significance:**

These findings suggest that PKD2L1 partly contributes to sour taste responses in mice and that receptors other than PKDs would be involved in sour detection.

## Introduction

Sour taste serves to detect acids in foods and drinks to deter the animals from ingesting spoiled and unripe food sources [1]. Though many candidate sour taste receptors have been implicated in detection, such as acid sensing ion channels (ASICs; [2]), hyperpolarization activated cyclic nucleotide gated potassium channels (HCNs; [3]), potassium channels [4], [5], NPPB sensitive Cl^−^ channels [6], and polycystic kidney disease 1L3 and 2L1 heteromers (PKD1L3+PKD2L1, [7], [8]), none of them has a demonstrated role in sour taste sensation in loss of function animals. PKD2L1 is a potential candidate because genetic elimination of cells expressing PKD2L1 substantially reduces gustatory nerve responses to sour taste stimuli [8]. Patients with sour ageusia (taste blind), but not normal subjects, lack the expression of mRNA for PKD1L3 and PKD2L1 (and ASIC1a, 1β, 2a, 2b and 3 as well) in the anterior part of the tongue [9], raising the possibility that PKDs may have a role in sour taste sensation in humans.

PKD2L1 is a member of the transient receptor potential (TRP) superfamily of ion channels and PKD1L3 is a TRP related molecule [10]. PKD1 and PKD2 heteromer association is required for the formation of a functional receptor channel [11]. In heterologous expression systems, cells expressing both PKD1L3 and PKD2L1 responded specifically to solutions containing acids with a characteristic “off” response; activation starts when acid solution is removed [7], [12]. In taste tissue, PKD2L1 is coexpressed with cell type markers for Type III cells such as 5-HT, NCAM and PGP9.5 [13], and Type III cells respond to sour taste stimuli [14], [15]. Although PKD2L1 is expressed both in the fungiform papillae (FP) on the anterior part of the tongue and in the circumvallate papillae (CV) on the posterior part of the tongue, expression of PKD1L3 was only observed in the CV [7], [8], indicating that PKD1L3 may not function in the anterior tongue.

To determine the role of PKD2L1 and PKD1L3 in sour taste, we produced gene knock-out (KO) mice and analyzed gustatory nerve responses and taste cell responses. We found that PKD2L1^−/−^ mice showed 25∼45% of reduction in responses to sour stimuli in the anterior tongue but not in the posterior tongue, indicating that PKD2L1 contributes to sour taste detection in the anterior tongue. Small reduction of sour taste responses in anterior tongue and no reduction in the posterior tongue of mice lacking PKD2L1 indicate the contribution of sour receptors other than PKD2L1 for sour detection.

## Materials and Methods

### Animals

**Figure 1:**
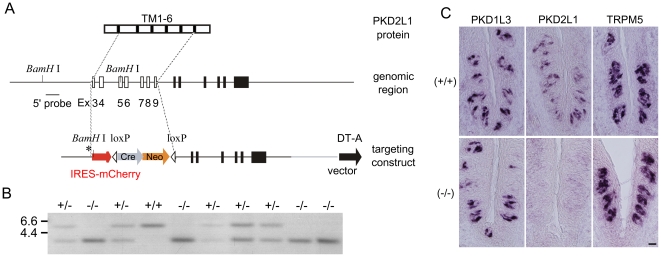
Generation of PKD2L1 knock-out mice. **A.** Schematic representation showing the structure of the PKD2L1 gene and the strategy for generating knock-out mice. The targeting construct deleted predicted transmembrane (TM) motifs 1 to 6. Ex: exon; Cre: Cre recombinase gene; Neo: neomycin resistant gene; loxP: loxP site; DT-A: diphtheria toxin A-chain gene. **B.** Genomic Southern blot analysis of PKD2L1^+/+^, PKD2L1^+/−^, and PKD2L1^−/−^ mice. BamHI-digested genomic DNAs extracted from wild-type, heterozygote, or homozygote mice were subjected to Southern blot analysis with the 5′-flanking probe that distinguishes wild type and deletion alleles for PKD2L1. **C.** *In situ* hybridization experiments demonstrating complete loss of PKD2L1 expression in the taste buds of the circumvallate papillae of PKD2L1^−/−^ mice and robust expression in wild-type mice. Scale bar, 20 µm.

All animal experiments were approved by the Animal Care and Use Committees at Kyushu University (A21-035-2), The University of Tokyo (P07-130), and Duke University (A217-10-09). Several lines of adult mice of both sexes (>8 week old) were used in this study; including PKD1L3 KO (PKD1L3^−/−^), PKD2L1 KO (PKD2L1^−/−^), PKD1L3/PKD2L1 double KO (PKD1L3/2L1^dbl−/−^), PKD1L3/PKD2L1 double wild type (WT), GAD67-GFP transgenic (GAD-GFP+WT), PKD2L1 KO with GAD67-GFP transgenic (GAD-GFP+PKD2L1^−/−^) and PKD1L3 KO with GAD67-GFP transgenic (GAD-GFP+PKD1L3^−/−^) mice. PKD1L3^−/−^ mice, in which the genomic region that contains exons 26 to 31, encoding transmembrane (TM) motif 7 to 11 of PKD1L3, was replaced by internal ribosomal entry site (IRES)-enhanced green fluorescent protein (EGFP) followed by the loxP sequence, were described previously [16]. The strategy for generating PKD2L1^−/−^ mice is illustrated in Fig. 1A. In the KO mice, the genomic region that contains exons 3 to 9, encoding TM motif 1 to 6 of PKD2L1, was replaced by IRES-mCherry and followed by the loxP sequence. Fragments used for the left and right arms were amplified by PCR using a BAC clone (RP24-224B6) (BACPAC Resources Center, Oakland, CA), derived from C57BL/6 mice as a template. The neomycin resistance gene in the ACN cassette [17] and the diphtheria toxin A-chain (DT-A) gene were used for positive and negative selection, respectively. The targeting vector was electroporated into the EF1 embryonic stem cell line [kindly provided by Dr. Frederick W. Alt (Harvard Medical School, Boston, MA, USA)], a 129SvEv/C57B6 hybrid, and the colonies were selected in G418-containing medium. Genomic DNA was digested by BamHI and hybridized with a 500-bp external probe on Southern blots. Two targeted ES cell clones were injected into C57BL/6 blastocysts, and chimeric mice were bred with C57BL/6 mice. The PCR primers for genotyping were 5′-ttctggtccagtttgctcag-3′ and 5′-catcaagtcccaggagtcaa-3′ for the wild-type allele and 5′-ttgatctgcaatgcaatgaacc-3′ and 5′-ccttattccaagcggcttcggccagtaacg-3′ for the mutated allele. PKD1L3^−/−^ mice were crossed to PKD2L1^−/−^ mice to bred PKD1L3^+/−^/PKD2L1^+/−^ mice. These mice were interbred to generate PKD1L3^−/−^, PKD2L1^−/−^, PKD1L3/2L1^dbl−/−^ and WT mice. Generation and characterization of GAD67-GFP mice have been described [18]. These mice were back-crossed to PKD2L1^−/−^ mice or PKD1L3^−/−^ mice to bred GAD-GFP+PKD2L1^−/−^ mice or GAD-GFP+PKD1L3^−/−^ mice.

### Solutions

Taste solutions used in this study were the following (in mM): 100 NH_4_Cl, 1∼20 HCl, 1∼50 citric acid, 1∼100 acetic acid, 10∼1000 sucrose, 10∼500 NaCl, 0.1∼10 quinine-HCl, 10∼1000 monosodium glutamate (MSG), 10∼1000 monopotasium glutamate (MPG). All of these chemicals were dissolved in distilled water (DW) at room temperature (20∼25°C). In taste cell recording, Tyrode solution was used as the extracellular solution. Tyrode solution contained (in mM): NaCl, 140; KCl, 5; CaCl_2_, 1; MgCl_2_, 1; HEPES, 10; Glucose, 10; sodium pyruvate, 10; pH adjusted to 7.4 with NaOH. All of these chemicals were purchased from Wako Pure Chemical Industries (Osaka, Japan).

### Gustatory nerve recordings

Gustatory nerve responses to lingual application of tastants were recorded from the chorda tympani (CT) or the glossopharyngeal (GL) nerve as described [19]. Under pentobarbital anesthesia (50∼60 mg/Kg b.w.), the trachea of each mouse was cannulated and the mouse was then fixed in the supine position with a head holder to allow dissection of the CT or the GL nerve. The right CT nerve was dissected free from surrounding tissues after removal of the pterygoid muscle and cut at the point of its entry to the bulla. The right GL nerve was exposed by removal of the digastricus muscle and posterior horn of the hyoid bone. The GL nerve was then dissected free from underlying tissues and cut near its entrance to the posterior lacerated foramen. For whole nerve recordings, the entire nerve was placed on the Ag/AgCl electrode. An indifferent electrode was placed in nearby tissue. Neural activities were fed into an amplifier (K-1; Iyodenshikagaku, Nagoya, Japan), and monitored on an oscilloscope and audiomonitor. Whole nerve responses were integrated with a time constant 1.0 sec and recorded on a computer using a PowerLab system (PowerLab/sp4; AD Instrument, Australia).

For taste stimulation of FP, the anterior one-half of the tongue was enclosed in a flow chamber made of silicone rubber. For taste stimulation of CV and foliate papillae, an incision was made on each side of the animal's face from the corner of the mouth to just above the angle of the jaw, and the papillae were exposed and their trenches opened by slight tension applied through a small suture sewn in the tip of the tongue. Taste solutions were delivered to each part of the tongue by gravity flow and flowed over the tongue for 30 sec (CT) or 60 sec (GL). The tongue was washed with DW for an interval of ∼1 min between successive stimulation. Only responses from stable recordings were used in data analysis.

### Taste cell recordings

The procedures for recording of taste cell responses were similar to those used previously [15], [20]. Subjects were GAD-GFP mice or GAD-GFP+PKD2L1^−/−^ mice. Animals were anesthetized with ether and sacrificed by cervical dislocation. The anterior part of the tongue was removed and injected with 100 µl of Tyrode solution containing 0.2∼1 mg/ml elastase (Elastin Products, Owensville, MO). After incubation for 10∼15 min at room temperature, the lingual epithelium was peeled and pinned out in a Sylgard coated culture dish with the mucosal side down and washed several times with Tyrode solution. Individual fungiform taste buds with a piece of epithelium were excised from this sheet and transferred to a recording chamber. The residual sheet was stored at 4°C for another series of experiments.

The recording chamber containing excised taste buds was mounted on the stage of a laser scanning confocal microscope (FV-1000 and Fluoview; Olympus, Tokyo, Japan). The mucosal side of an excised epithelium with single taste bud was drawn into the orifice of the stimulating pipette. Tyrode solution was continuously flowed into the recording chamber with a peristaltic pump at approximately 2 ml/min. The receptor membrane was rinsed with DW for at least 30 sec before and after taste stimulation. The electrical responses of taste cells in isolated taste buds were recorded extracellularly from the basolateral side at room temperature (25°C). Taste bud cells containing GFP were identified under confocal laser scanning microscopy (excitation 488 nm, emission 500∼600 nm) and were approached by a recording electrode (2.5∼4 MΩ). Seal resistances were typically 3∼10 times the pipette resistances. Electrical signals from taste bud cells were recorded by a high-impedance patch-clamp amplifier (Axopatch 200B; Axon Instruments, Foster City, CA) interfaced to a computer (Windows XP) by an analog-to-digital board (Digidata 1320A; Axon Instruments). Signals were filtered at 1 KHz, sampled at 5∼10 KHz and stored on the hard-disk drive of a computer using pCLAMP software (Gap-Free mode; Axon Instruments) for later analysis.

### Data analysis

In the analysis of whole nerve responses, integrated whole-nerve response magnitudes were measured 5, 10, 15, 20, and 25 sec (for the CT) and 5, 10, 20, 30, and 40 sec (for the GL) after stimulus onset, averaged, and normalized to responses to 100 mM NH_4_Cl to account for mouse to mouse variations in absolute responses. This relative response was used for statistical analysis (two-way ANOVA and the post hoc Dunnett's test, or t-test). In the analysis of taste cell responses, action potential waveforms were analyzed in respect to the following parameters: time of peak-peak, peak amplitude/antipeak amplitude ratio, antipeak amplitude, and peak amplitude [21]. The number of spikes per unit time was counted throughout the recording. The mean spontaneous impulse discharge for each unit was calculated by averaging the number of spikes over the 10 sec period in which distilled water flowed over the taste pore prior to each stimulation. The magnitude of response to taste stimulus was obtained by counting the total number of impulses for the first 10 sec after the onset of stimulus application and subtracting the spontaneous impulse discharge. We used data from single taste cells that were defined by the following criteria: 1) the number of spikes evoked by 10 mM HCl or 10 mM citric acid was larger than the mean plus 2 standard deviations of the spontaneous discharge in two repeated trials and 2) at least +3 spikes were evoked by 10 mM HCl or 10 mM citric acid.

### RT-PCR

Mouse taste buds in peeled epithelium were individually removed from the FP and CV by aspiration with a transfer pipette. 30 taste buds from the taste papillae or 1×1 mm block of the epithelial tissue without taste buds were used to purify RNAs by RNeasy Plus Mini Kit (Qiagen, Hilden, Germany). Reverse transcription (RT) and first round amplification took place in the same tube using a OneStep RT-PCR kit (Qiagen) according to the manufacturer's instructions. A 50 µl reaction mixture contained the following: 10 µl Qiagen OneStep RT-PCR buffer (×5), 2 µl Qiagen OneStep RT-PCR enzyme mix, 0.4 mM of each dNTP, 1 µl RNase inhibitor, 0.2∼0.6 mM of each outside primers (Table 1) and the sample (1/30 of purified RNAs). After the RT reaction at 50°C for 30 minutes, the first round of PCR was subsequently performed in the same tube with a 15 minute preincubation at 95°C followed by 30 cycles of denaturation (94°C for 30 sec), annealing (53°C for 60 sec), and amplification (72°C for 90 sec) in a thermal cycler (TaKaRa PCR thermal cycler: Takara, Tokyo, Japan). Subsequently, the first round PCR products were re-amplified for 35 cycles (94°C for 30 sec, 60°C for 30 sec, 72°C for 60 sec) in separate reactions using the internal primer pairs for each template. Each 10 µl second round reaction mix contained the following: 0.25 Units of Taq DNA polymerase (TaKaRa Ex Taq™ HS: Takara), 1 µl of 10× PCR buffer containing 20 mM Mg^2+^, 0.2 mM of each dNTP, 0.6 mM of each internal primer pair (Table 1) and 0.2 µl of first round PCR products. After a second round of amplification, reaction solutions were subjected to 2% agarose gel electrophoresis with ethidium bromide. Negative control reactions were run in parallel from the RT-PCR. β-actin was used as internal control. All primer sets were designed to span exon-intron boundaries to distinguish PCR products derived from genomic DNA and mRNA.

**Table 1:** Nucleotide sequences of primers used in RT-PCR experiments (Horio et al.).

Gene	Accession No.	Forward	Reverse	Product size
Gustducin	NM_001081143	ACGAGATGCAAGAACTGTGA	TATCTGTCACGGCATCAAAC	941 bp
		TGCTTTGAAGGAGTGACGTG	GTAGCGCAGGTCATGTGAGA	341 bp
PKD1L3	NM_181544	ACGGTCTTCAATGCTAATGT	ATAACCTCCTTGTGCTTTGA	671 bp
		AAAAGGAACCTCCTGGACAC	CCAAACAGCAGGTTGAAAGT	347 bp
PKD2L1	NM_181422	CCCTGTGTACTTTGTCACCT	GTGACACCTAGGACGGATTA	680 bp
		CTTCACCAGGTTTGATCAGG	TTCCTCTCCAGCATCTTCAG	300 bp
β-actin	NM_007393	CCTGAAGTACCCCATTGAAC	GTAACAGTCCGCCTAGAAGC	943 bp
		GGTTCCGATGCCCTGAGGCTC	ACTTGCGGTGCACGATGGAGG	370 bp

Upper: outside primers.

Lower: inside primers.

### *In situ* hybridization and immunohistochemistry

*In situ* hybridization was performed essentially as described [7]. The probe for PKD1L3 contained the region encoding from TM2 to the C-terminus (V1176-Y2151). The probes for PKD2L1 and TRPM5 contained the entire coding regions. For immunohistochemical staining, the dissected tongues of mice were fixed in 4% paraformaldehyde in PBS for 30∼90 min at 4°C. After dehydration with sucrose solution (10% for 1 h, 20% for 1 h, 30% for 3 h at 4°C), the frozen block of fixed tongue was embedded in OCT compound (Sakura Finetechnical, Tokyo, Japan), sectioned into 9-µm-thick slices, which were mounted on silane-coated glass slides. Frozen sections were washed with TNT buffer, treated with 1% blocking reagent (Roche, Mannheim, Germany) for 1 h at room temperature, and incubated overnight at 4°C with primary antibodies for PKD1L3 (goat polyclonal IgG; Lifespan Bioscience, WA, USA), PKD2L1 [7] or GAD67 (goat polyclonal IgG; SantaCruz, CA, USA) in 1% blocking reagent. After washing with TNT buffer, tissues were incubated for 2 h at room temperature with secondary antibodies (Alexa Fluor 555, donkey anti-rabbit IgG; Invitrogen, OR, USA) in 1% blocking reagent. The immunofluorescence of labeled taste cells and GFP fluorescence were observed by using a laser scanning microscope (FV-1000, Olympus, Tokyo, Japan) and images were obtained by using Fluoview software (Olympus). We counted positive cells in each taste bud in horizontal sections of FP and CV [22]. Image-ProPlus (Ver. 4.0, Maryland, USA) was used to exclude artifactual signals: cells showing a density signal greater than the mean plus two standard deviations of the density in taste cells in the negative control (primary antibody was omitted) were considered positive. The same cells found on the contiguous sections were counted only once.

## Results

### PKD1L3^−/−^, PKD2L1^−/−^ and PKD1L3/2L1^dbl−/−^ mice

**Figure 2:**
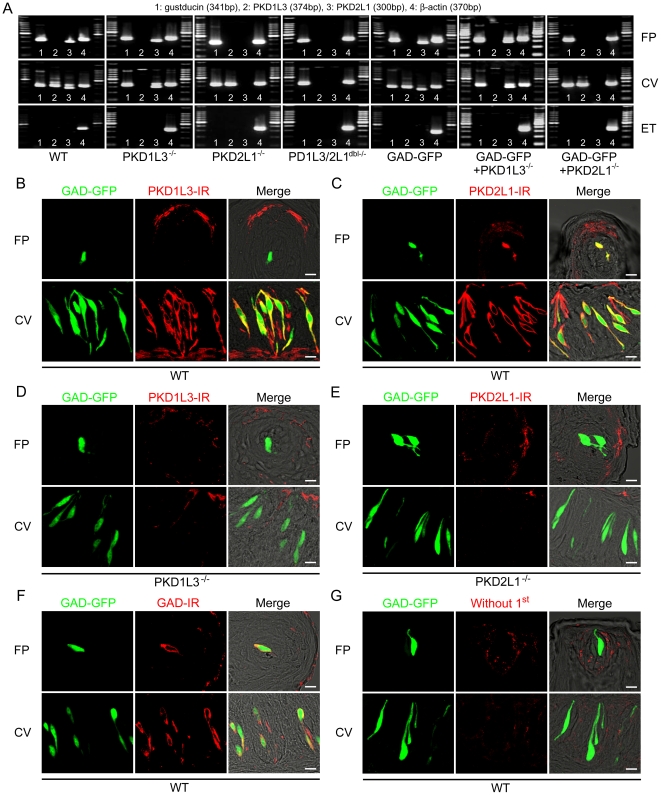
Expression of PKDs in taste tissues. **A.** mRNA expression of PKD1L3 and PKD2L1 in taste tissues of WT, PKD1L3^−/−^, PKD2L1^−/−^, PKD1L3/2L1^dbl−/−^, GAD-GFP, GAD-GFP+PKD1L3^−/−^, and GAD-GFP+PKD2L1^−/−^ mouse. FP: fungiform taste buds. CV: circumvallate taste buds. ET: epithelial tissue. Gustducin is a control for taste tissue. β-actin is an internal control. 100 bp marker was used. **B**, **C.** Immunostaining for PKD1L3 (**B**) and PKD2L1 (**C**) in taste tissues of GAD-GFP+WT mouse. **D.** Immunostaining for PKD1L3 in taste tissues of GAD-GFP+PKD1L3^−/−^ mouse. **E.** Immunostaining for PKD2L1 in taste tissues of GAD-GFP+PKD2L1^−/−^ mouse. **F.** Immunostaining for GAD67 in taste tissue of GAD-GFP+WT mouse. **G.** Immunostaining without primary antibody in GAD-GFP+WT mouse. Green shows GFP fluorescence. Red shows immunoreactivity (IR) for PKD1L3, PKD2L1 or GAD67, respectively. FP: fungiform papillae. CV: circumvallate papillae. Scale bar, 10 µm.

We first generated several lines of gene KO mice. We used PKD1L3^−/−^ mice that were described in a previous study [16]. We generated PKD2L1 ^−/−^ mice lacking the predicted transmembrane (TM) motifs 1 to 6 (Fig. 1A). Genomic Southern blot analysis shows typing of PKD2L1^+/+^, PKD2L1^+/−^, and PKD2L1^−/−^ mice (Fig. 1B). The lack of expression of PKD2L1, but not the sweet/umami/bitter taste cell marker TRPM5, in CV of PKD2L1^−/−^ mice was confirmed by *in situ* hybridization (Fig. 1C). PKD1L3^−/−^ mice or PKD2L1^−/−^ mice have knockin of GFP or mCherry ([16], Fig. 1A) but GFP or mCherry fluorescence arising from this construct was not observed in these strains of mice. We crossed PKD1L3^−/−^ mice and PKD2L1^−/−^ mice to produce PKD1L3^+/−^ and PKD2L1^+/−^ mice. These mice were then intercrossed to yield PKD1L3^−/−^, PKD2L1^−/−^, PKD1L3/2L1^dbl−/−^, and WT mice. Previous studies using *in situ* hybridization demonstrated that PKD2L1 expression was observed in both FP and CV, whereas PKD1L3 expression was detected in the CV but not in the FP [7], [8]. We examined mRNA expression of PKDs in the FP and CV of WT, PKD1L3^−/−^, PKD2L1^−/−^, and PKD1L3/2L1^dbl−/−^ mice (Fig. 2A). Consistent with previous reports, the expression of PKD2L1 was detected in both the FP and CV and the expression of PKD1L3 was observed in only the CV of WT mice (Fig. 2A). PKD1L3^−/−^ mice or PKD2L1^−/−^ mice lacked the expression of PKD1L3 or PKD2L1 in either papillae, respectively (Fig. 2A). PKD1L3/2L1^dbl−/−^ mice did not express both PKD1L3 and PKD2L1 in both papillae (Fig. 2A). Thus, each type of KO mice lacked the expression of mRNA for PKD1L3 or/and PKD2L1. PKD2L1 is coexpressed with Type III cell markers such as 5-HT, NCAM and PGP9.5 but not with Type I and II cell markers such as NTPD2ase, PLCβ2, and TRPM5 [13]. Because GAD67 is expressed in a subset of type III cells [23], [24], and almost all of the GFP-positive cells in GAD67-GFP mice would be positive for GAD67 (Fig. 2F, Table 2, [18]), we produced GAD-GFP−PKD2L1^−/−^ mice and GAD-GFP−PKD1L3^−/−^ mice then examined the expression pattern of GFP and PKD2L1 or PKD1L3 in the FP and CV. In RT-PCR, mRNA expression of PKD1L3 and PKD2L1 in GAD-GFP mice was the same as that in WT mice (Fig. 2A), while GAD-GFP+PKD2L1^−/−^ mice or GAD-GFP+PKD1L3^−/−^ mice lacked mRNA expression of PKD2L1 or PKD1L3 in both papillae, respectively (Fig. 2A). In immunohistochemical experiments, GFP positive cells in the CV of GAD-GFP mice were positive for both PKD1L3 and PKD2L1 whereas GFP positive cells in the FP of GAD-GFP mice expressed PKD2L1 but not PKD1L3 (Fig. 2B, C). Also, some GAD-GFP negative cells showed the immunoreactivity for PKD1L3 or PKD2L1. PKD1L3^−/−^ mice or PKD2L1^−/−^ mice lacked the expression of PKD1L3 or PKD2L1 in both papillae, respectively (Fig. 2D, E). These results indicate that PKD2L1 is expressed in both the FP and CV whereas PKD1L3 is expressed only the CV of WT mice and that PKD1L3^−/−^ mice or PKD2L1^−/−^ mice lack protein expression of PKD1L3 or PKD2L1 in taste tissues.

**Table 2:** Summary of immunohistochemical data on PKD2L1, PKD1L3, and GAD67 (Horio et al.).

	WT				PKD2L1^−/−^				PKD1L3^−/−^			
	FP	%	CV	%	FP	%	CV	%	FP	%	CV	%
PKD2L1-IR/GAD67-GFP	45/47	95.7	164/165	99.4	0/45	0	0/143	0	-	-	-	-
PKD1L3-IR/GAD67-GFP	0/62	0	151/156	96.8	-	-	-	-	0/46	0	0/153	0
GAD67-IR/GAD67-GFP	41/43	95.3	153/156	98.1	44/45	97.8	220/223	98.7	36/38	94.7	143/144	99.3

Numbers represent numbers of cells.

-: not examined.

2∼3 animals were used for each data.

### Whole nerve responses

**Figure 3:**
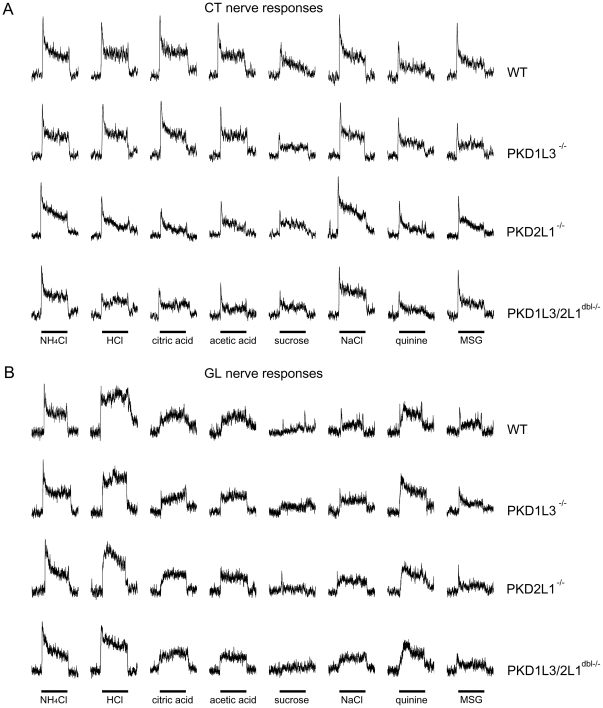
Sample recordings of gustatory nerve responses of WT, PKD1L3^−/−^, PKD2L1^−/−^, and PKD1L3/2L1^dbl−/−^ mice. **A.** CT nerve responses. **B.** GL nerve responses. Taste stimuli were NH_4_Cl (100 mM), HCl (10 mM), citric acid (10 mM), acetic acid (30 mM), sucrose (500 mM), NaCl (100 mM), quinine (10 mM), MSG (100 mM). Bars indicate taste stimulation (30 sec for CT nerve responses; 60 sec for GL nerve responses).

**Figure 4:**
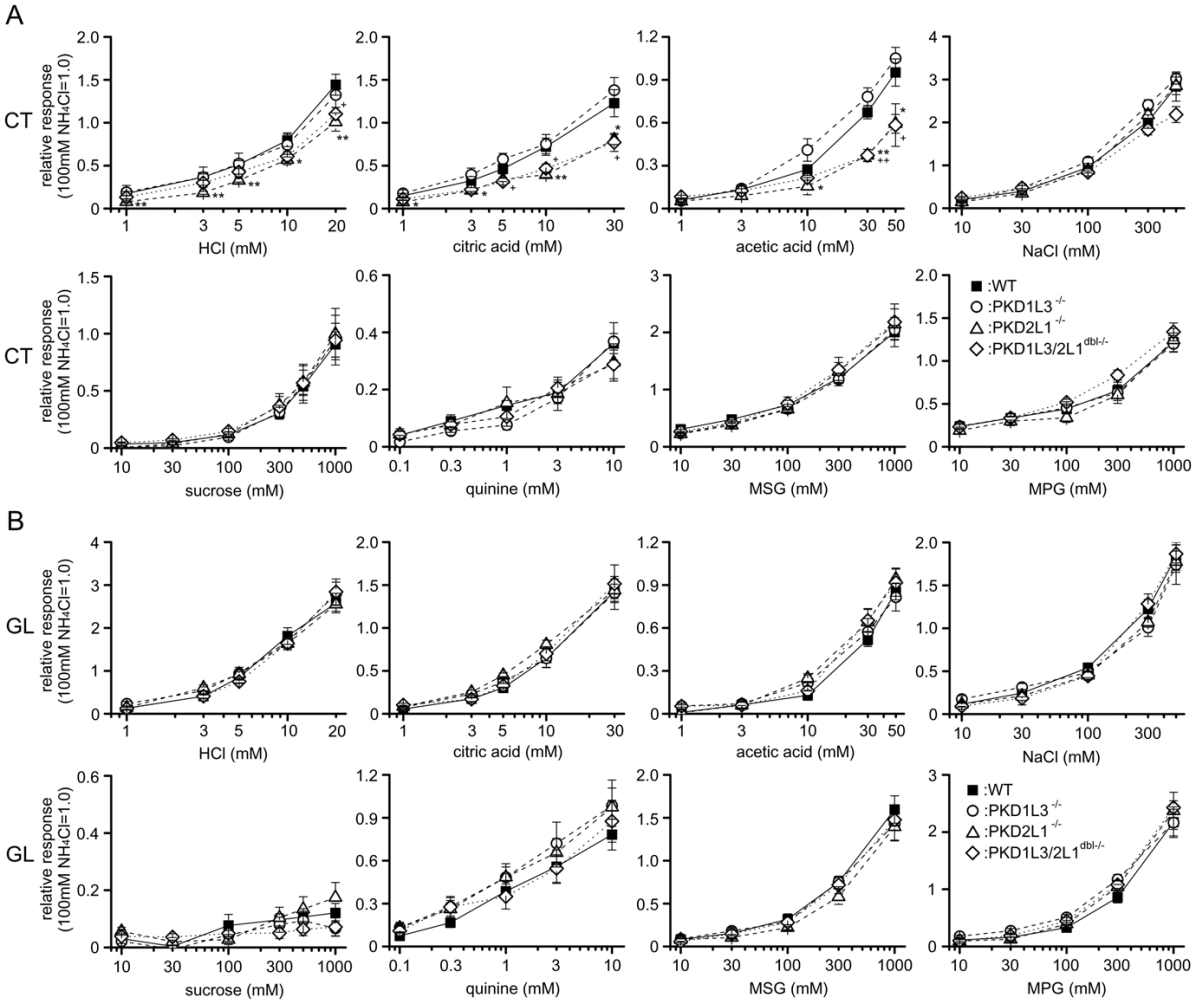
Concentration-response relationships of CT and GL nerve responses. Concentration-response relationships of CT (**A**) and GL (**B**) nerve responses of WT [black rectangle, n = 8 (CT) and 8 (GL)], PKD1L3^−/−^ [white circle, n = 5 (CT), and 6 (GL)], PKD2L1^−/−^ [white triangle, n = 7 (CT) and 6 (GL)], and PKD1L3/2L1^dbl−/−^ mice [white diamond, n = 6 (CT) and 6 (GL)] for HCl, citric acid, acetic acid, NaCl, sucrose, quinine, MSG, and MPG. Gustatory nerve responses were normalized to the response to 100 mM NH_4_Cl. Values indicated are means ± S.E.M. Statistical differences were analyzed by ANOVA tests (see Table 3) and *post hoc* Dunnett's tests (*: P<0.05, **: P<0.01 for PKD2L1^−/−^; ^+^: P<0.05, ^++^: P<0.01 for PKD1L3/2L1^dbl−/−^).

Using the gene KO and control mice, we recorded chorda tympani (CT) and glossopharyngeal (GL) nerve responses to taste stimuli. The CT and GL nerves relay the gustatory information in the anterior and posterior parts of the tongue, respectively. All types of mice used in this study showed significant whole nerve responses to sweet (sucrose), salty (NaCl), bitter (quinine), umami (monosodium glutamate; MSG which also contains the Na^+^ component and monopotasium glutamate; MPG which contains the K^+^ component) and sour (HCl, citric acid, acetic acid) tastants in both the CT and the GL nerve (Fig. 3, 4). CT nerve responses to sour tastants in PKD2L1^−/−^ and PKD1L3/2L1^dbl−/−^ mice were significantly smaller than those in WT mice (∼55–75% of WT responses; Fig. 3A and 4A). CT nerve responses to different concentrations of HCl, acetic acid, and citric acid were significantly reduced in PKD2L1^−/−^ and PKD1L3/2L1^dbl−/−^ mice but not in PKD1L3^−/−^ mice (ANOVA and post hoc Dunnett's test; Fig. 4A, Table 3). CT nerve responses to different concentrations of sweet, salty, bitter, and umami compounds and GL nerve responses to different concentrations of all tastants tested were not significantly different between KO and WT mice (ANOVA test; Fig. 3, 4, Table 3). These results suggest that PKD2L1 is specifically involved in sour taste transduction in the anterior tongue but not in the posterior tongue.

**Table 3:** ANOVA results for CT and GL nerve responses to taste compounds (vs. WT mice) (Horio et al.).

		PKD1L3^−/−^		PKD2L1^−/−^		PKD1L3/2L1^dbl−/−^	
nerve	tastant	DF	F	DF	F	DF	F
CT	HCl	1,64	0.2	1,74	30.1***	1,69	12.1***
	CA	1,64	1.6	1,74	20.9***	1,69	14.5***
	AA	1,64	2.4	1,74	22.5***	1,69	12.0***
	Suc	1,64	2.1	1,74	0.4	1,69	1.4
	NaCl	1,64	2.0	1,74	0.0	1,69	2.8
	QHCl	1,64	1.5	1,74	1.3	1,69	3.8
	MSG	1,64	0.2	1,74	0.0	1,69	0.3
	MPG	1,64	0.0	1,74	0.7	1,69	2.9
GL	HCl	1,69	0.7	1,69	0.0	1,69	0.0
	CA	1,69	0.2	1,69	3.8	1,69	0.6
	AA	1,69	0.9	1,69	3.8	1,69	2.9
	Suc	1,69	1.7	1,69	0.6	1,69	2.3
	NaCl	1,69	0.6	1,69	2.2	1,69	0.2
	QHCl	1,69	3.8	1,69	3.4	1,69	0.4
	MSG	1,69	0.3	1,69	3.4	1,69	0.7
	MPG	1,69	2.6	1,69	0.7	1,69	2.7

Response magnitudes were analyzed by two-way ANOVA. Table based on data shown in Fig. 4. DF: degree of freedom. F: F values.

***: P<0.001, ANOVA.

### Taste cell responses

**Figure 5:**
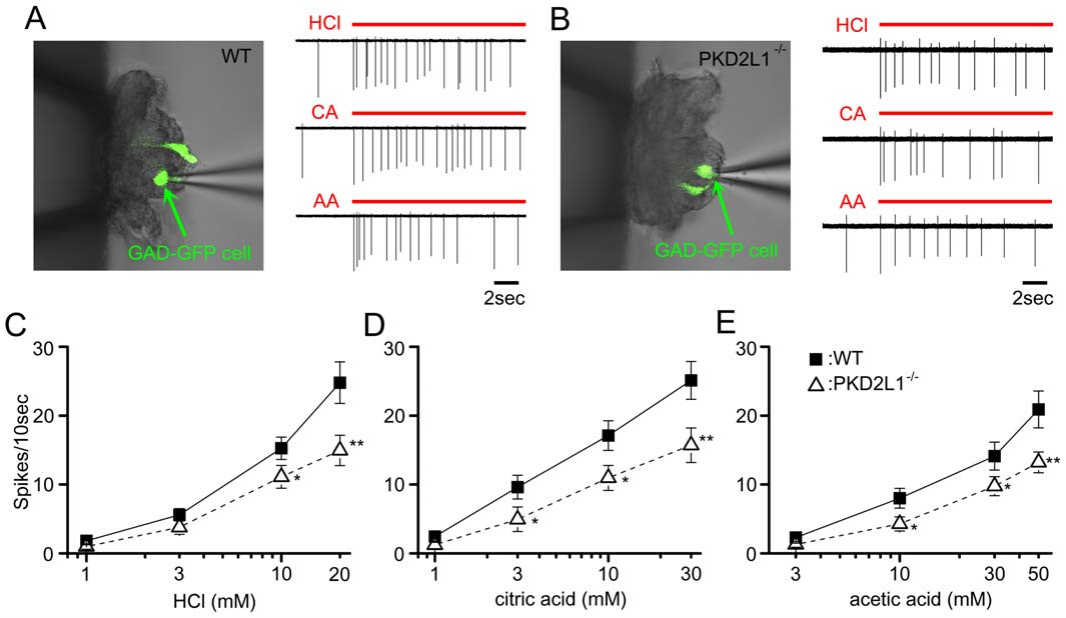
Taste responses of GAD67-GFP taste cells in FP taste buds of WT and PKD2L1^−/−^ mice. **A**, **B.** Sample recording from a GAD67-GFP taste cell (see picture) of WT (**A**) and PKD2L1^−/−^ (**B**) mice. Taste responses to 10 mM HCl, 10 mM citric acid (CA), and 30 mM acetic acid (AA) were shown. Red bars indicate duration of taste stimulation. **C–E.** Concentration-response relationships of taste responses of GAD67-GFP taste cells for HCl (**C**), citric acid (**D**) and acetic acid (**E**). Taste responses of PKD2L1^−/−^ mice (white triangle, HCl: n = 18∼27, citric acid: n = 17∼24, acetic acid: n = 17∼24) were significantly smaller than those of WT mice (black rectangle, HCl: n = 17∼31, citric acid: n = 16∼30, acetic acid: n = 17∼25) in ANOVA tests (see Result) and *post hoc t*-tests (*: P<0.05, **: P<0.01). Values indicated are means ± S.E.M.

PKD2L1 is coexpressed with GAD67-GFP taste cells of WT mice (Fig. 2C). Our previous study demonstrated that GAD67-GFP taste cells in fungiform taste buds responded well to sour taste stimuli [15]. To examine whether PKD2L1 mediates sour taste responses in fungiform taste cells, we analyzed the taste responses of GAD67-GFP taste cells in fungiform taste buds of WT and PKD2L1^−/−^ mice. Sample recordings from GAD67-GFP taste cells of WT and PKD2L1^−/−^ mice (Fig. 5A,B) showed that both taste cells responded well to three sour tastants (HCl, citric acid and acetic acid), although responses of GAD67-GFP taste cells of the PKD2L1^−/−^ mouse were smaller than those of the WT mouse. In total, 45/143 (33.8%) of GFP taste cells of WT mice and 35/126 (27.8%) of GFP taste cells of PKD2L1^−/−^ mice responded to sour taste stimuli. We further examined responses of GAD67-GFP taste cells to various concentrations of sour compounds (Fig. 5C∼E). The magnitude of sour taste responses of GAD67-GFP taste cells depended on the concentration of sour tastants applied. Though GAD67-GFP cells of PKD2L1^−/−^ mice responded to sour taste stimuli, the responses in PKD2L1^−/−^ mice were significantly decreased than those in WT mice in ANOVA analysis [F_(1,169)_ = 12.1, P<0.01 for HCl; F_(1,161)_ = 16.3, P<0.01 for citric acid; F_(1,160)_ = 12.8, P<0.01 for acetic acid]. These results suggest that PKD2L1 is at least partly involved in sour taste transduction in GAD67 expressing taste cells of the FP.

## Discussion

Genetic elimination of cells expressing PKD2L1 substantially reduces CT nerve responses to sour taste stimuli [8]. Here we for the first time demonstrated that mice lacking PKD2L1 had diminished but not abolished CT nerve responses and FP taste cell responses to sour tastants (Fig. 3∼5), suggesting that PKD2L1 contributes to sour taste detection in mice. Because PKD2L1 KO mice appear to show less severe defects in acid-evoked CT nerve responses compared to mice in which cells expressing PKD2L1 were eliminated, we predict that molecules other than PKD2L1 also function in sour transduction in the anterior tongue. We found no significant change in GL nerve responses in PKD2L1 gene knockouts as well as PKD2L1+PKD1L3 double knockouts, suggesting that these molecules may not function in taste transduction in the posterior tongue. Together, sour taste transduction may rely on multiple receptor systems, analogous to amiloride-sensitive and -insensitive receptors for salt taste [25]–[27], and T1r3-dependent and -independent pathways for sweet and umami taste [19], [28] although the effect of PKD2L1 knock-out on sour detection may be smaller than that of ENaC knock-out on salt taste detection and T1R knock-out on sweet and umami detection. Thus, the receptor system for each taste quality may consist of multiple taste receptors.

In humans, patients with an acquired sour ageusia that were unresponsive to sour stimuli and showed normal responses to bitter, sweet and salty stimuli lacked the expression of not only PKD1L3 and PKD2L1 but ASIC1a, 1β, 2a, 2b and 3 in FP taste buds as well [9]. Therefore, responses to sour stimuli in PKD2L1^−/−^ mice may be mediated by ASIC subunits. In human and rat, ASIC2 is expressed in taste tissue [2], [9] and is implicated as a sour taste receptor. However, this subunit is absent in the taste buds of mice and Ca^2+^ responses of taste cells to sour taste stimuli were unaltered in ASIC2-KO mice [29]. Instead, ASIC1 and 3 are expressed in mouse taste buds [29] and may function as sour taste receptors. HCN4, another sour receptor candidate, is coexpressed with PKD2L1 [30] and may play a role in sour taste detection although cesium, an inhibitor of HCN currents, did not affect taste cell responses to sour stimuli [31]. A recent study demonstrated that responses to acids are mediated by a proton conductance but not by Na^+^ permeable channels specific to PKD2L1 expressing CV taste cells [32]. Such H^+^ specific conductance in PKD2L1 expressing taste cells, which may be mediated by NADPH-dependent and cAMP-sensitive H^+^ channels [33], would contribute to sour taste responses without PKD2L1. Intracellular acidification is another possible route for sour taste detection in taste cells [14], [31], [34], [35]. Mouse taste buds express K_2_P channels such as TWIK-1 and -2, TREK-1 and -2, and TASK-1 [4], [5]. These channels are blocked by intracellular (and extracellular) acidification [36], which leads to a depolarization of taste cells. Pharmacological blockers for these channels affect acid responses of taste cells [5], suggesting that K_2_P channels contribute to sour taste responses in taste cells. In addition, application of 5-nitro2-(3-phenylpropylamino)-benzoic acid (NPPB) blocks responses to citric acid in nondissociated taste cells from the FP [6], suggesting the involvement NPPB sensitive Cl^−^ channels in sour reception. Our results showing remaining responses to sour tastants in PKD KO mice indicate that one or more of these candidates may be involved in sour taste detection in taste cells. However, further studies are needed to reveal the *in vivo* function and/or molecular identity of these candidates.

Our results demonstrated that the lack of PKD1L3 had no significant effect on CT and GL nerve responses to all tastants tested (Fig. 3, 4). A recent report shows similar results [37]. PKD1L3 and PKD2L1 are coexpressed in the same subset of taste cells of the CV [38], although PKD1L3 is not expressed in mouse FP [7], [8], consistent with our observation that CT nerve responses were not affected in PKD1L3^−/−^ mice. Both PKD1L3 and PKD2L1 interact with each other and are necessary for forming a functional channel at the cell surface [7], [16], similar to PKD1 and PKD2 [11]. Indeed, HEK cells expressing both PKD1L3 and PKD2L1 showed robust Ca^2+^ responses to sour stimuli but HEK cells expressing PKD1L3 or PKD2L1 alone did not [7], [12], [39]. These responses occurred at the end of acid stimulation, termed an off-response [12]. Off-responses have been reported to be induced by sour stimuli in mammals [40]–[42], and this was also shown in the isolated taste cells of the CV [43]. Taken together, PKD1L3 and PKD2L1 may function by inducing off-responses but not contribute to on-responses to sour stimuli in the posterior part of the tongue. In contrast, the lack of PKD2L1 reduced on-responses to sour stimuli in the anterior part of the tongue (Fig. 3∼5). Solo expression of PKD2L1 in HEK cells did not induce sour responses [7], [12], [39], suggesting that PKD2L1 may need an unidentified partner that is expressed in the FP but not in the CV to mediate on-responses to sour stimuli. Topographical difference between the FP and the CV was also seen in gustatory nerve responses to sour taste stimuli. CT nerve responses were smaller than control (responses to 100 mM NH_4_Cl), while GL nerve responses were larger than control (Fig. 3, 4). No comparable observation was seen for other tested acids. Such differences may imply the topographic segregation of sour taste receptors on the tongue.

In conclusion, we found that PKD2L1 at least partly contributes to sour taste responses at the level of FP taste cells and whole CT nerves. Future work is necessary to identify the whole set of receptor and transduction systems for sour taste including ones that partner with PKD2L1.
